# Home blood pressure-lowering effect of esaxerenone vs trichlormethiazide for uncontrolled hypertension: a prespecified subanalysis of the EXCITE-HT randomized controlled study by age subgroup

**DOI:** 10.1038/s41440-024-02078-8

**Published:** 2025-03-28

**Authors:** Kazuomi Kario, Hiroyuki Ohbayashi, Masami Hashimoto, Naoki Itabashi, Mitsutoshi Kato, Kazuaki Uchiyama, Kunio Hirano, Noriko Nakamura, Takahide Miyamoto, Hirotaka Nagashima, Hidenori Ishida, Yusuke Ebe, Tsuguru Hatta, Toshiki Fukui, Tomohiro Katsuya, Tatsuo Shimosawa, Takashi Taguchi, Ayumi Tanabe, Mitsuru Ohishi, on behalf of the EXCITE-HT investigators

**Affiliations:** 1 Division of Cardiovascular Medicine, Department of Medicine, Jichi Medical University School of Medicine, Shimotsuke, Tochigi Japan; 2 Tohno Chuo Clinic, Mizunami, Gifu Japan; 3 Hashimoto Kidney Clinic, Fukuyama, Hiroshima Japan; 4 Itabashi Diabetes and Dermatology Medical Clinic, Koga, Ibaraki Japan; 5 Kato Clinic of Internal Medicine, Katsushika-ku, Tokyo Japan; 6 Uchiyama Clinic, Joetsu, Niigata Japan; 7 Hirano Clinic, Morioka, Iwate Japan; 8 Primula Clinic, Kagoshima, Kagoshima Japan; 9 Miyamoto Clinic of Internal Medicine, Matsumoto, Nagano Japan; 10 Tokyo Center Clinic, Chuo-ku, Tokyo Japan; 11 Akaicho Clinic, Chiba, Chiba Japan; 12 Ebe Clinic, Nagaoka, Niigata Japan; 13 Hatta Medical Clinic, Kyoto, Kyoto Japan; 14 Olive Takamatsu Medical Clinic, Takamatsu, Kagawa Japan; 15 Katsuya Clinic, Amagasaki, Hyogo Japan; 16 Department of Clinical Laboratory, School of Medicine, International University of Health and Welfare, Narita, Chiba Japan; 17 Primary Medical Science Department, Medical Affairs Division, Daiichi Sankyo Co., Ltd., Chuo-ku, Tokyo Japan; 18 Data Intelligence Department, Daiichi Sankyo Co., Ltd., Shinagawa-ku, Tokyo Japan; 19 Department of Cardiovascular Medicine and Hypertension, Graduate School of Medical and Dental Sciences, Kagoshima University, Kagoshima, Kagoshima Japan

**Keywords:** Age, Esaxerenone, Essential hypertension, Trichlormethiazide

## Abstract

This predefined subanalysis of the multicenter, randomized, open-label, parallel-group EXCITE-HT study aimed to determine whether the comparative efficacy and safety of esaxerenone and trichlormethiazide differs with age. Patients were divided into two age subgroups (<65 and ≥65 years). The non-inferiority of esaxerenone to trichlormethiazide was assessed based on the upper limit of the two-sided 95% confidence interval (CI) for the difference in systolic/diastolic blood pressure (SBP/DBP) changes. Esaxerenone was considered non-inferior if this value was <3.9/ < 2.1 mmHg; if it was <0 mmHg, esaxerenone was considered superior in its BP-lowering effect. The results showed that the least squares mean changes in morning home SBP/DBP from baseline to the end of treatment (primary endpoint) were −9.5/−5.7 with esaxerenone and −8.2/−4.9 mmHg with trichloromethiazide (between-group difference: −1.3 [95% CI, −3.3, 0.8]/−0.8 [ − 2.1, 0.5] mmHg) in the subgroup aged <65 years. These changes were −14.6/−7.2 and −11.5/−6.7 (−3.0 [−4.9, −1.2]/−0.5 [−1.5, 0.5] mmHg) in the subgroup aged ≥65 years. The incidences of serum potassium level ≥5.5 mEq/L were 2.2% and 1.9% in the esaxerenone-treated subgroups aged <65 and ≥65 years, respectively. In conclusion, esaxerenone achieved the pre-defined non-inferiority margin to trichlormethiazide in its BP-lowering effect regardless of age. In patients aged <65 years, esaxerenone achieved the non-inferiority margin to trichlormethiazide in lowering both SBP and DBP. In patients aged ≥65 years, esaxerenone was superior to trichlormethiazide in lowering SBP and achieved the non-inferiority margin to trichlormethiazide in lowering DBP. The impact of esaxerenone on serum potassium levels did not show a specific age-related effect.

**Figure:**
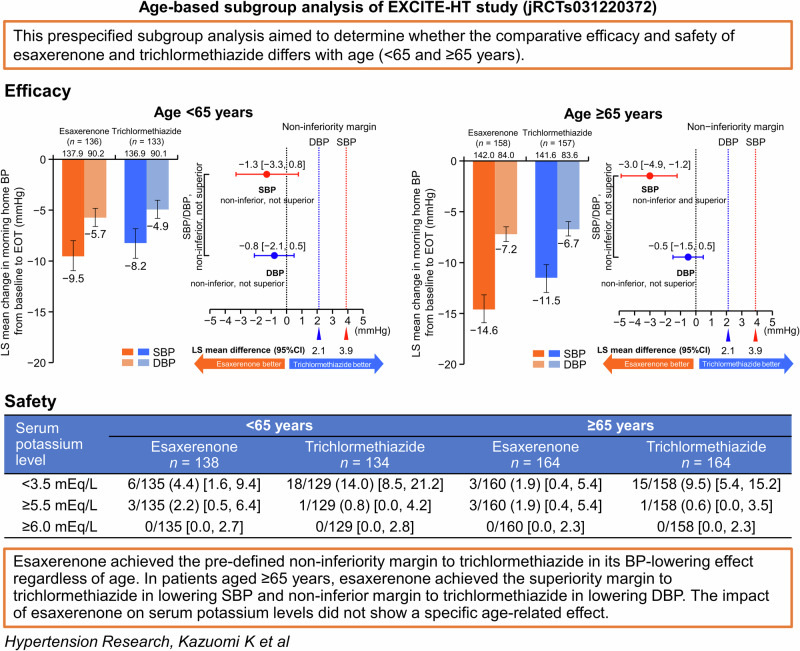
A subgroup analysis of the EXCITE-HT study according to age (<65 and ≥65 years) showed that esaxerenone achieved the pre-defined non-inferiority margin to trichlormethiazide in its BP-lowering effect regardless of age. In patients aged ≥65 years, esaxerenone achieved the superiority margin to trichlormethiazide in lowering SBP

A subgroup analysis of the EXCITE-HT study according to age (<65 and ≥65 years) showed that esaxerenone achieved the pre-defined non-inferiority margin to trichlormethiazide in its BP-lowering effect regardless of age. In patients aged ≥65 years, esaxerenone achieved the superiority margin to trichlormethiazide in lowering SBP

## Abstract (web summary)

A subgroup analysis of the EXCITE-HT study according to age (<65 and ≥65 years) showed that esaxerenone achieved the pre-defined non-inferiority margin to trichlormethiazide in its BP-lowering effect regardless of age. In patients aged ≥65 years, esaxerenone achieved the superiority margin to trichlormethiazide in lowering SBP.

## Introduction

In Japan, the growing aging population has led to a high prevalence of hypertension [1, 2]. Appropriate long-term blood pressure (BP) control, including morning home BP, is critical to prevent cardiovascular and cerebrovascular events [3–8], especially in older people [9–12]. Therefore, there is an urgent need to extend healthy life expectancy through the prevention and control of hypertension.

Despite the availability of a wide range of antihypertensive agents, optimal BP control is not always achieved in some patients [13, 14]. A possible reason for this phenomenon, known as the “hypertension paradox” [15–17], might be the limited efficacy of single-agent antihypertensive therapy; thus, a muti-drug approach may be necessary to achieve optimal BP control in some patients, including older patients. Treatment of hypertension in older patients requires careful consideration of comorbidities and the potential risk of adverse events (AEs) due to polypharmacy [18]. Furthermore, older patients have an increased risk of developing salt-sensitive hypertension [19, 20] and decreased renin activity [21], making BP control more challenging and underscoring the need for effective and safe combination antihypertensive therapy.

In the 2019 Japanese Society of Hypertension (JSH) guidelines [22], the selection of antihypertensive agents—including combination therapy—and antihypertensive targets are the same for older and younger patients. The first-line treatments recommended by the JSH 2019 guidelines, such as calcium channel blockers (CCBs), renin–angiotensin system inhibitors (angiotensin II receptor blockers [ARBs] and angiotensin-converting enzyme inhibitors), and diuretics (thiazide diuretics and loop diuretics), are often prescribed to older hypertensive patients, taking into account any concomitant medications, comorbidities, or contraindications [22]. In Japan, ARBs and CCBs are the most frequently prescribed antihypertensive agents [23, 24].

Esaxerenone is a next-generation non-steroidal mineralocorticoid receptor blocker (MRB) that has a higher selectivity and potency, longer half-life, and more favorable bioavailability than other MRBs [25, 26]. The ESAX-HTN study demonstrated that the BP-lowering effect of esaxerenone (2.5 mg/day) was non-inferior to that of eplerenone (50 mg/day) [27]. In terms of safety, older age is a risk factor for hyperkalemia, and MRBs, including esaxerenone, cause hyperkalemia as a class effect [28].

The EXCITE-HT study demonstrated the non-inferiority of esaxerenone to trichlormethiazide in lowering morning home BP in patients with uncontrolled essential hypertension receiving an ARB or CCB [29, 30]. However, it remains unclear whether the comparative efficacy and safety of esaxerenone and trichlormethiazide differ with age. This EXCITE-HT study subgroup analysis, predefined in the statistical analysis plan, aimed to analyze age-related differences in the efficacy and safety of esaxerenone. We hypothesized that diuretics would be beneficial for patients with salt-sensitive hypertension [31] and that MRBs would be effective for older patients with salt-sensitive hypertension and low renin activity [32, 33]. This age-specific analysis was conducted to evaluate these hypotheses.

## Methods

### Study design

The EXCITE-HT study was a multicenter (54 sites), randomized, open-label, parallel-group study conducted between December 2022 and September 2023, the details of which have been reported previously [29, 30]. In this prespecified subgroup analysis of the EXCITE-HT study, patients were divided into two age subgroups (<65 and ≥65 years).

The primary study protocol was approved by the Certified Review Board of Hattori Clinic (CRB3180027) and was registered at the Japan Registry of Clinical Trials under the identifier jRCTs031220372 (https://jrct.niph.go.jp/en-latest-detail/jRCTs031220372). The study was conducted in accordance with the principles of the Declaration of Helsinki and the Clinical Trials Act in Japan. Written informed consent was obtained from patients prior to enrollment.

### Patients

The EXCITE-HT study included patients aged ≥20 years who received previous treatment with either one ARB or one CCB at the same dose for ≥4 weeks before registration and who had mean morning home systolic BP (SBP) ≥ 125 mmHg and/or diastolic BP (DBP) ≥ 75 mmHg. Patients aged ≥75 years with cerebrovascular disease or proteinuria-negative chronic kidney disease were eligible if they had a mean morning home SBP ≥ 135 mmHg and/or DBP ≥ 85 mmHg [29, 30].

### Study interventions

Esaxerenone (starting dose: 2.5 mg/day and 1.25 mg/day in patients with estimated glomerular filtration rate [eGFR] 30–59 mL/min/1.73 m^2^ or in those with diabetes mellitus and albuminuria or proteinuria at baseline; maximum dose, 5 mg/day) was administered for 12 weeks according to the Japanese package insert [34]. Doses could be gradually increased to 5 mg/day based on BP and serum potassium level after 4 or 8 weeks of treatment. Trichlormethiazide was administered at the discretion of the treating physician according to the Japanese package insert and JSH 2019 guidelines [22, 35], in which the recommended starting dose is ≤1 mg/day. The dose could be increased after 4 or 8 weeks of treatment at the physician’s discretion in line with the patient’s condition. Basal antihypertensive agents (ARBs or CCBs) were given at a constant dosage throughout the treatment period until the end of treatment (EOT); the use of other antihypertensive agents was prohibited.

### Study endpoints

The primary endpoint was the change in morning home SBP/DBP from baseline to EOT. The secondary endpoints were the change in bedtime home and office SBP/DBP from baseline to EOT and the change in urinary albumin-to-creatinine ratio (UACR) and serum N-terminal pro-brain natriuretic peptide (NT-proBNP) levels from baseline to Week 12. The safety endpoints were as follows: the time course changes and change from baseline in eGFR and serum potassium throughout the study period; the proportion of patients with serum potassium level ≤3.5 mEq/L, ≥5.5 mEq/L, and ≥6.0 mEq/L; and the proportion of patients with uric acid (UA) level >7.0 mg/dL. The methods for measuring BP have been reported previously [29].

### Sample size and statistical analyses

The target sample size was determined for the primary study and not specifically for this subgroup analysis. Data were analyzed by age subgroups (<65 and ≥65 years). The statistical methods used in the primary analysis were also used for this subgroup analysis [29, 30]. Briefly, if the upper limit of the two-sided 95% confidence interval (CI) for the difference in both SBP and DBP change between esaxerenone and trichlormethiazide was <3.9 mmHg and <2.1 mmHg, respectively, esaxerenone was deemed non-inferior to trichlormethiazide in its BP-lowering effect. If the upper limit of the two-sided 95% CI was <0 mmHg, esaxerenone was deemed superior to trichlormethiazide in its BP-lowering effect. The main analysis was conducted using the full analysis set (FAS), and an ancillary analysis was conducted using the per-protocol set (PPS). All statistical analyses were conducted with a two-sided significance level of 5%, unless otherwise stated. The statistical software used was SAS version 9.4 or higher (SAS Institute Inc., Cary, NC, USA).

## Results

### Patients

In the primary study, 600 patients were eligible and randomly assigned to the esaxerenone and trichlormethiazide groups (295 and 290 patients, respectively, in the FAS; 275 and 290 patients, respectively, in the PPS) [30]. Among patients in the FAS, the subgroup aged <65 years included 137 and 133 patients in the esaxerenone and trichlormethiazide groups, respectively, and the subgroup aged ≥65 years included 158 and 157 patients in the esaxerenone and trichlormethiazide groups, respectively.

Baseline patient demographic and clinical characteristics are shown in Table 1. The mean morning home SBP was numerically higher and the mean morning home DBP was numerically lower in the subgroup aged ≥65 years vs <65 years (<65 years: 137.9/90.2 and 136.9/90.1 mmHg in the esaxerenone and trichlormethiazide groups, respectively; ≥65 years: 142.0/84.0 and 141.6/83.6 mmHg in each treatment group, respectively). Similar trends were observed in the mean office SBP/DBP (<65 years: 141.2/87.9 and 140.1/88.3 mmHg in each treatment group, respectively; ≥65 years: 146.1/79.6 and 145.0/79.3 mmHg in each treatment group, respectively). The mean bedtime home SBP/DBP was similar between age subgroups (<65 years: 133.7/84.6 and 134.0/85.4 mmHg in each treatment group, respectively; ≥65 years: 135.5/78.7 and 134.8/77.7 mmHg in each treatment group, respectively).

**Table 1:** Baseline patient characteristics (full analysis set)

Characteristics	<65 years		≥65 years	
	Esaxerenone *n* = 137	Trichlormethiazide *n* = 133	Esaxerenone *n* = 158	Trichlormethiazide *n* = 157
Sex, male	74 (54.0)	82 (61.7)	75 (47.5)	78 (49.7)
Age, years	54.9 ± 6.7	54.0 ± 7.9	74.2 ± 6.1	73.9 ± 6.1
Weight, kg	72.95 ± 14.76	73.16 ± 14.08	59.35 ± 10.97	61.10 ± 11.10
Body mass index, kg/m^2^	27.04 ± 4.92	26.86 ± 4.66	23.94 ± 3.07	24.41 ± 3.38
Morning home SBP, mmHg	137.9 ± 14.8	136.9 ± 12.5	142.0 ± 15.0	141.6 ± 13.3
Morning home DBP, mmHg	90.2 ± 9.7	90.1 ± 9.2	84.0 ± 8.7	83.6 ± 8.4
Bedtime home SBP, mmHg	133.7 ± 16.0 *n* = 133	134.0 ± 13.6	135.5 ± 15.7 *n* = 148	134.8 ± 14.4 *n* = 149
Bedtime home DBP, mmHg	84.6 ± 10.6 *n* = 133	85.4 ± 10.1	78.7 ± 9.5 *n* = 148	77.7 ± 10.2 *n* = 149
Office SBP, mmHg	141.2 ± 14.0	140.1 ± 15.3	146.1 ± 18.1	145.0 ± 14.7
Office DBP, mmHg	87.9 ± 10.7	88.3 ± 11.6	79.6 ± 11.0	79.3 ± 11.2
NT-proBNP, pg/mL	51.75 ± 99.88 *n* = 104	53.80 ± 166.14 *n* = 116	154.64 ± 397.27 *n* = 133	109.29 ± 124.43 *n* = 137
<55	76 (73.1)	93 (80.2)	49 (36.8)	47 (34.3)
55 to <125	21 (20.2)	17 (14.7)	42 (31.6)	55 (40.1)
≥125	7 (6.7)	6 (5.2)	42 (31.6)	35 (25.5)
UACR, mg/gCr	103.50 ± 583.89	96.87 ± 563.19	127.33 ± 374.21	105.15 ± 255.49
<30	97 (70.8)	98 (73.7)	96 (60.8)	89 (56.7)
30 to <300	35 (25.5)	28 (21.1)	48 (30.4)	55 (35.0)
≥300	5 (3.6)	7 (5.3)	14 (8.9)	13 (8.3)
Serum potassium, mEq/L	4.16 ± 0.36 *n* = 133	4.18 ± 0.32 *n* = 127	4.25 ± 0.34 *n* = 153	4.24 ± 0.32 *n* = 151
Uric acid, mg/dL	5.52 ± 1.38	5.66 ± 1.15	5.27 ± 1.14	5.19 ± 1.22 *n* = 156
eGFR_creat_, mL/min/1.73 m^2^	76.60 ± 15.84	77.27 ± 15.61 *n* = 131	67.07 ± 14.28	67.82 ± 17.14 *n* = 156
Duration of hypertension, years	3.88 ± 4.26 *n* = 85	4.41 ± 4.13 *n* = 86	6.59 ± 5.40 *n* = 99	6.38 ± 5.57 *n* = 98
Complication	131 (95.6)	121 (91.0)	154 (97.5)	155 (98.7)
T2DM	50 (36.5)	49 (36.8)	69 (43.7)	66 (42.0)
Dyslipidemia	86 (62.8)	71 (53.4)	102 (64.6)	100 (63.7)
Hyperuricemia	29 (21.2)	32 (24.1)	16 (10.1)	12 (7.6)
Heart failure	2 (1.5)	3 (2.3)	20 (12.7)	13 (8.3)
Esaxerenone dose at baseline (initial dose), mg				
1.25	39 (28.5)	-	79 (50.0)	-
2.5	98 (71.5)	-	79 (50.0)	-
Esaxerenone dose at EOT (last dose), mg				
1.25	19 (13.9)	-	46 (29.1)	-
2.5	82 (59.9)	-	92 (58.2)	-
5	36 (26.3)	-	20 (12.7)	-
Trichlormethiazide dose at baseline (initial dose), mg				
0.25	-	2 (1.5)	-	2 (1.3)
0.5	-	10 (7.5)	-	7 (4.5)
1	-	117 (88.0)	-	145 (92.4)
2	-	4 (3.0)	-	3 (1.9)
Trichlormethiazide dose at EOT (last dose), mg				
0.25	-	2 (1.5)	-	0
0.5	-	7 (5.3)	-	11 (7.0)
1	-	109 (82.0)	-	135 (86.0)
>1 to ≤2	-	13 (9.8)	-	11 (7.0)
≥3	-	2 (1.5)	-	0
Basal antihypertensive agent				
Angiotensin receptor blocker	56 (40.9)	56 (42.1)	63 (39.9)	60 (38.2)
Calcium channel blocker	81 (59.1)	77 (57.9)	95 (60.1)	97 (61.8)

Data are n (%) or mean ± standard deviation

*DBP* diastolic blood pressure, *eGFR*_*creat*_ creatinine-based estimated glomerular filtration rate, *EOT* end of treatment, *NT-proBNP* N-terminal pro-brain natriuretic peptide, *SBP* systolic blood pressure, *T2DM* type 2 diabetes mellitus, *UACR* urinary albumin-to-creatinine ratio

The baseline diabetes complication rate and UACR were numerically higher in the subgroup aged ≥65 years than in the subgroup aged <65 years. The baseline eGFR was numerically lower and serum potassium level were numerically higher in the subgroup aged ≥65 years than in the subgroup aged <65 years. The mean UACR was 103.50 and 96.87 mg/gCr in the esaxerenone and trichlormethiazide treatment groups, respectively, in the subgroup aged <65 years and 127.33 and 105.15 mg/gCr in each treatment group, respectively, in the subgroup aged ≥65 years. The mean serum potassium level was 4.16 and 4.18 mEq/L in each treatment group, respectively, in the subgroup aged <65 years and 4.25 and 4.24 mEq/L in each treatment group, respectively, in the subgroup aged ≥65 years. The mean eGFR was 76.60 and 77.27 mL/min/1.73 m^2^ in each treatment group, respectively, in the subgroup aged <65 years and 67.07 and 67.82 mL/min/1.73 m^2^ in each treatment group, respectively, in the subgroup aged ≥65 years. There were no notable differences in the use of basal antihypertensive agents (ARB and CCB) between the two age subgroups.

Among patients who received esaxerenone, the proportion of patients who received a dose of 5 mg as the last dose was lower in the subgroup aged ≥65 years than in the subgroup aged <65 years (12.7% vs 26.3%, respectively). Among patients who received trichlormethiazide, the most common last dose was 1 mg and the proportion of patients who received this dose was higher in the subgroup aged ≥65 years than in the subgroup aged <65 years (86.0% vs 82.0%, respectively). Baseline patient demographic and clinical characteristics of the PPS are shown in Supplementary Table 1.

### BP-lowering effects

**Fig. 1:**
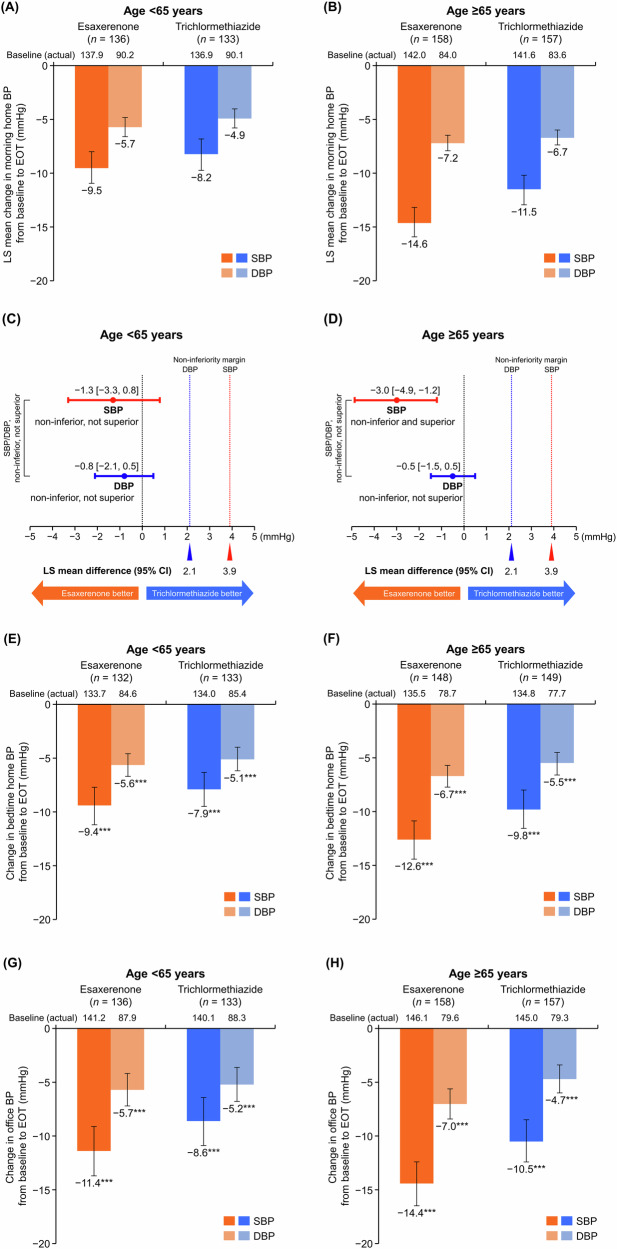
Changes from baseline to EOT in (**A**, **B**, **C, D**) morning home, (**E**, **F**) bedtime home, and (**G**, **H**) office BP (full analysis set). **(A**, **C**, **E**, **G)** Subgroup aged <65 years; (**B**, **D**, **F**, **H**) subgroup aged ≥65 years. For (**C**, **D**), the red dotted line (3.9 mmHg) and blue dotted line (2.1 mmHg) indicate the non-inferiority criteria. Data are LS mean (95% CI) for (**A**–**D**). Data are arithmetic mean (95% CI) for (**E**–**H**). ***P < 0.001 versus baseline, paired *t*-test. *BP* blood pressure, *CI* confidence interval, *DBP* diastolic blood pressure, *EOT* end of treatment, *LS* least squares, *SBP* systolic blood pressure

Morning home SBP/DBP significantly decreased from baseline to EOT in all subgroups (Supplementary Table 2). The least squares (LS) mean changes in morning home SBP/DBP from baseline to EOT were −9.5 (95% CI, −10.9, −8.0)/−5.7 (−6.6, −4.8) and −8.2 (−9.7, −6.8)/−4.9 (−5.8, −4.0) mmHg in the esaxerenone and trichlormethiazide groups, respectively, within the subgroup aged <65 years (Fig. 1A) and −14.6 (−15.9, −13.2)/−7.2 (−7.9, −6.5) and −11.5 (−12.9, −10.2)/−6.7 (−7.4, −6.0) mmHg, respectively, within the subgroup aged ≥65 years (Fig. 1B).

The between-group difference in LS mean change was −1.3 (95% CI, −3.3, 0.8)/−0.8 (−2.1, 0.5) mmHg in the subgroup aged <65 years (Fig. 1C) and −3.0 (−4.9, −1.2)/−0.5 (−1.5, 0.5) mmHg in the subgroup aged ≥65 years (Fig. 1D).

Significant reductions were also shown in bedtime home and office SBP/DBP in both age subgroups (all P < 0.001) (Fig. 1E–H and Supplementary Table 2). Similar results in morning home, bedtime home, and office BP measurement were shown in the PPS (Supplementary Table 3).

### UACR and NT-proBNP

**Fig. 2:**
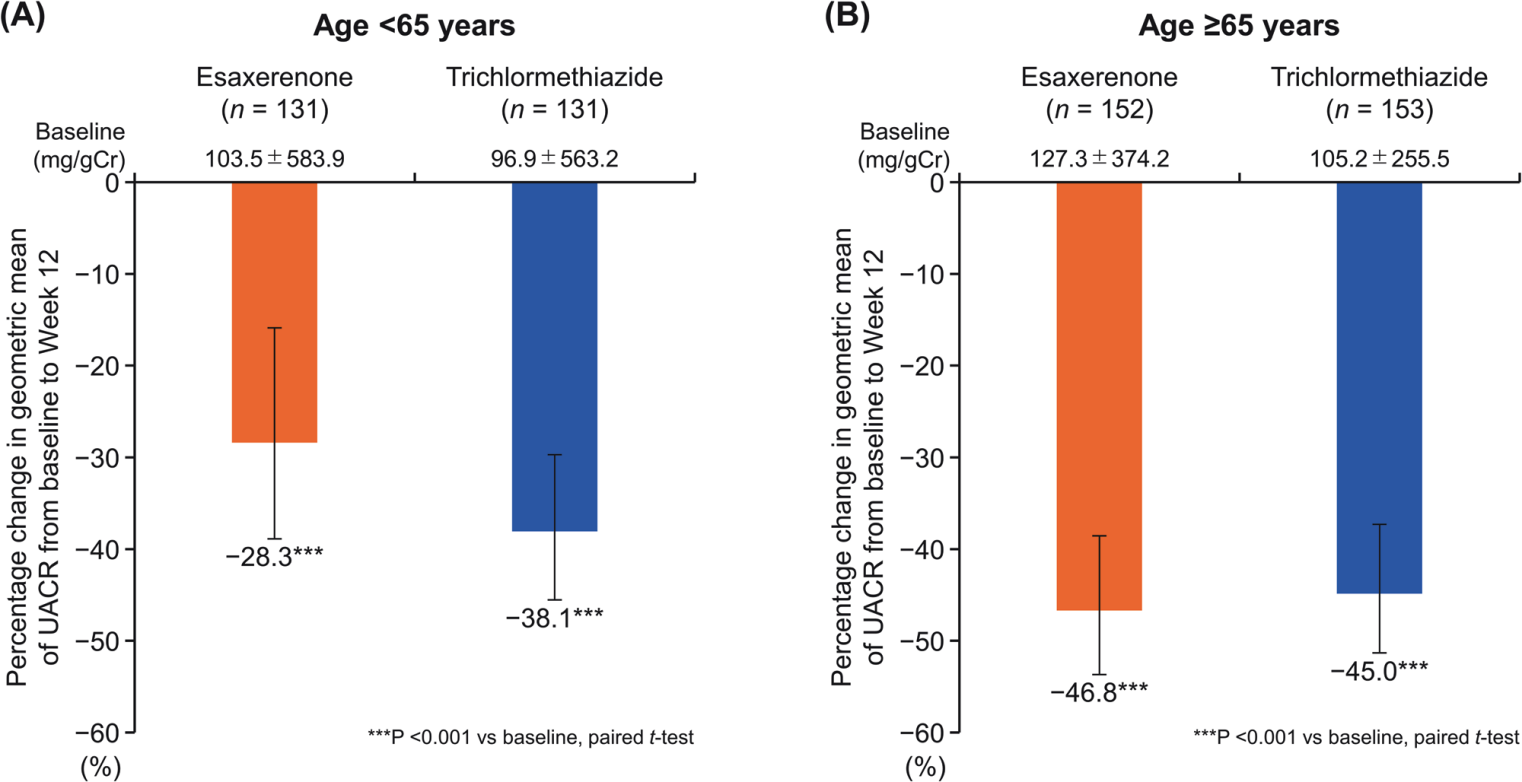
Percentage change in geometric mean of UACR during the study period (full analysis set). **(A)** Subgroup aged <65 years; (**B)** subgroup aged ≥65 years. Data are mean (95% CI). ***P < 0.001 versus baseline, paired *t*-test. *CI* confidence interval, *UACR* urinary albumin-to-creatinine ratio

The geometric mean of UACR significantly decreased from baseline to Week 12 in all subgroups (<65 years; −28.3% for esaxerenone, −38.1% for trichlormethiazide; ≥65 years: −46.8% for esaxerenone, −45.0% for trichlormethiazide; all *P* < 0.001 versus baseline) (Fig. 2A, B, Supplementary Table 4). Changes in NT-proBNP levels from baseline to Week 12 are shown in Supplementary Tables 4 and 5.

### eGFR, serum potassium, and UA levels

**Fig. 3:**
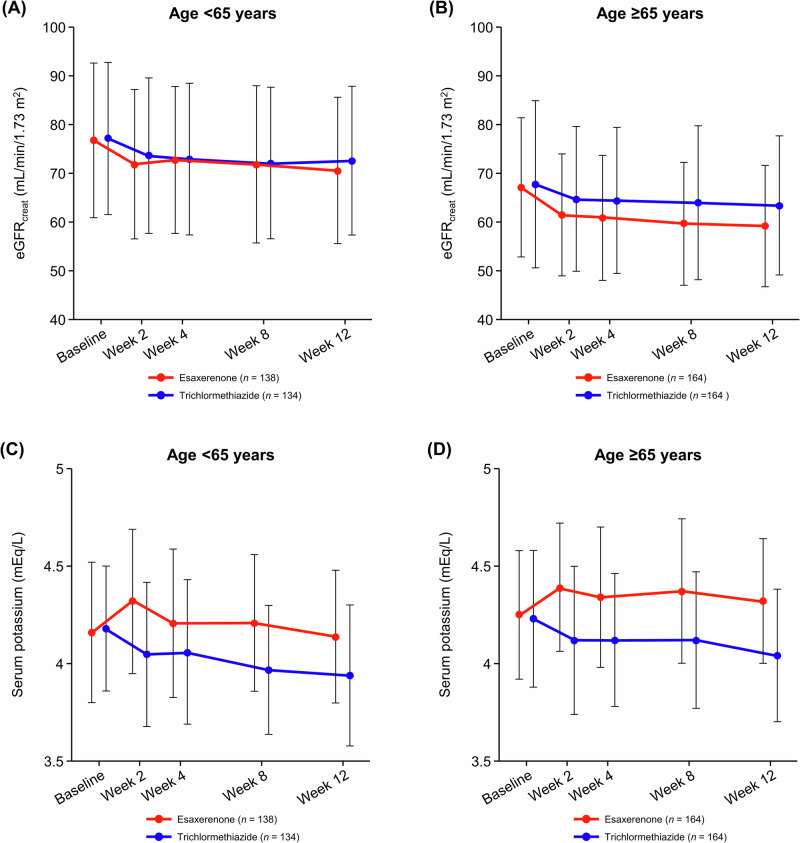
Time course changes in *eGFR*_*creat*_ (**A**, **B**) and serum potassium levels (**C**, **D**) during the study period (safety analysis set). (**A**, **C)** Subgroup aged <65 years; **(B**, **D)** subgroup aged ≥65 years. Data are mean ± SD. *eGFR*_*creat*_ creatinine-based estimated glomerular filtration rate, *SD* standard deviation

Time course changes in eGFR and serum potassium are shown in Fig. 3A–D. eGFR decreased over the first 2 weeks and remained relatively stable until Week 12 (Fig. 3A, B, and Supplementary Table 6). The change in eGFR from baseline to Week 12 was −6.00 ± 8.08 and −4.61 ± 8.98 mL/min/1.73 m^2^ in the esaxerenone and trichlormethiazide groups, respectively, within the subgroup aged <65 years and −8.02 ± 8.96 and −4.73 ± 10.54 in each treatment group, respectively, within the subgroup aged ≥65 years.

Serum potassium levels increased over the first 2 weeks after starting esaxerenone treatment in both age subgroups. Levels remained almost constant until Week 12 in the subgroup aged ≥65 years, whereas in the subgroup aged <65 years, after the first 2 weeks, serum potassium levels gradually decreased to Week 12 (Fig. 3C, D and Supplementary Table 6). Serum potassium levels gradually decreased in the trichlormethiazide group regardless of the age subgroup. The respective proportions of patients with serum potassium levels <3.5 mEq/L and ≥5.5 mEq/L were 4.4% and 2.2% in the esaxerenone group and 14.0% and 0.8% in the trichlormethiazide group within the subgroup aged <65 years; within the subgroup aged ≥65 years, these proportions were 1.9% each in the esaxerenone group and 9.5% and 0.6% in the trichlormethiazide group (Supplementary Table 7). No patients with serum potassium levels ≥6.0 mEq/L were observed in any subgroup.

The proportions of patients with UA levels >7.0 mg/dL were numerically higher in the trichlormethiazide group compared with the esaxerenone group in both subgroups aged <65 and ≥65 years (<65 years: esaxerenone, 33.3% and trichlormethiazide, 43.3%; ≥65 years: esaxerenone, 22.6% and trichlormethiazide, 27.4%; Supplementary Table 8).

## Discussion

### Summary of results

Until now, there have been limited papers reporting differences in the efficacy and safety of esaxerenone by age in a clinical setting, and this is the first report of such differences. In this subgroup analysis of the EXCITE-HT study, the percentage of patients aged ≥65 years was 53.8% (315/585); overall, the efficacy and safety results were similar between the two age subgroups. The non-inferiority of esaxerenone to trichlormethiazide in lowering morning home BP was confirmed regardless of age. In patients <65 years, esaxerenone achieved the pre-defined non-inferiority margin to trichlormethiazide in lowering both SBP and DBP, and in patients ≥65 years, esaxerenone was superior in lowering SBP and non-inferior in lowering DBP. Additionally, significant reductions from baseline were observed in bedtime home and office SBP/DBP in both age subgroups. The proportion of patients with serum potassium level ≥5.5 mEq/L was similar between both age groups among patients treated with esaxerenone, with no patients having a serum potassium level ≥6.0 mEq/L in any subgroup. In contrast, among patients treated with trichlormethiazide, the proportion of patients with serum potassium <3.5 mEq/L was higher in patients aged <65 years compared with those aged ≥65 years.

### Antihypertensive effect by age

The antihypertensive effect of esaxerenone showed superiority to trichlormethiazide in lowering SBP and non-inferiority to trichlormethiazide in lowering DBP in patients aged ≥65 years, which differed from that in patients aged <65 years. This is consistent with a previous phase 3 study of esaxerenone that showed a trend towards a greater BP-lowering effect in older patients and those with lower renin activity [27, 36].

At the start of this study, half of the patients aged ≥65 years received 1.25 mg of esaxerenone as a starting dose, and fewer patients in this subgroup had their dose increased to 5 mg compared with the younger subgroup. This lower rate of dose increase among the older subgroup may have been because of age-related decline in renin activity, which may have influenced the decision to avoid increasing the dose in this patient population [37, 38]. Although renin activity was not measured in this study, previous reports have indicated that older patients are less likely to benefit from ARBs as renin activity decreases [39–41]. In a previous study, the MRB eplerenone was shown to be effective in the treatment of essential hypertension in patients with low renin activity [42].

CCBs are frequently used as first-line antihypertensive agents; if the antihypertensive effect is insufficient, second-line ARBs are often used. Additional administration of esaxerenone in both older and younger patients with inadequate antihypertensive response to CCBs is expected to have further antihypertensive effects. Furthermore, among patients treated with esaxerenone, a higher percentage of patients aged ≥65 years had complications such as diabetes mellitus and higher UACR (≥300 mg/gCr) compared with patients aged <65 years (diabetes mellitus: 43.7% vs 36.5%, respectively; UACR ≥ 300 mg/gCr: 8.9% vs 3.6%, respectively). This suggests that the older subgroup may have included a larger proportion of patients with mineralocorticoid receptor-related hypertension [43] who were more likely to respond to MRBs.

### Effect on UACR

UACR decreased in both the esaxerenone and trichlormethiazide groups regardless of age, but the UACR-lowering effect was numerically greater in patients aged ≥65 years. However, it should be noted that this is a simple observation of the change in UACR over a 12-week period, and further studies with a larger number of patients and longer observation period are needed to clarify the difference in the improvement of UACR between esaxerenone and trichlormethiazide.

### Serum potassium

Esaxerenone is known to cause hyperkalemia, while trichlormethiazide can cause hypokalemia [28, 44]. Older patients are at risk of developing both of these adverse effects. Patients with serum potassium >5.0 mEq/L at enrollment were excluded from the EXCITE-HT study [29], and there was no notable difference in serum potassium levels at enrollment by age subgroup or by treatment group in this subanalysis. Among patients treated with esaxerenone, none had serum potassium ≥6.0 mEq/L, regardless of age.

Although older age is a risk factor for hyperkalemia during treatment with MRBs including esaxerenone [28], this risk can be safely managed with reduced dosing and regular serum potassium monitoring according to the package insert [34]. In fact, there was no age-related difference in the proportion of patients with serum potassium ≥5.5 mEq/L in this study.

In this study, physicians determined the dose of esaxerenone based on the patient’s condition such as serum potassium level and antihypertensive effect. Older patients had a low last dose of esaxerenone, and starting with a lower dose of esaxerenone provided an adequate antihypertensive effect while appropriately managing the risk of hyperkalemia. Serum potassium levels peaked at Week 2 and decreased thereafter in patients aged <65 years, whereas they increased at Week 2 and remained stable in patients aged ≥65 years. This discrepancy may be due to varying eGFR between the two age subgroups (mean eGFR was 76.60 mL/min/1.73 m^2^ in patients aged <65 years and 67.07 mL/min/1.73 m^2^ in patients aged ≥65 years). Compared with the esaxerenone group, serum potassium elevations in the trichlormethiazide group were less frequent, regardless of age. However, among patients treated with trichlormethiazide, low serum potassium (<3.5 mEq/L) was more frequent in patients aged <65 years compared with those aged ≥65 years. In addition to differences in eGFR, the possibility of primary aldosteronism cannot be excluded. Although the protocol excluded patients with secondary hypertension including primary aldosteronism, the possibility of including a certain number of patients with primary aldosteronism cannot be ruled out because a definitive diagnosis of primary aldosteronism was not required.

### UA elevation

Regardless of age, the proportions of patients with UA levels >7.0 mg/dL were numerically higher in the trichlormethiazide group vs the esaxerenone group, which is consistent with the DIME study in which diuretics significantly increased UA [45]. Other studies have also reported increased risk of hyperuricemia with diuretics such as trichlormethiazide [46, 47]. Furthermore, UA levels were numerically higher in patients aged <65 years compared with patients aged ≥65 years in both treatment groups. The younger subgroup had higher baseline UA level, hyperuricemia, and body mass index (BMI), which indicated that the younger subgroup included more obese patients than the older subgroup, although the complication rates of dyslipidemia and type 2 diabetes mellitus were lower in the younger subgroup than the older subgroup. An association between UA level and obesity has been previously reported [48, 49], and this might explain our results. In the primary EXICTE-HT study, no cases of hyponatremia or reduction in serum sodium level as an AE were reported in the esaxerenone group and no increased risk of hyponatremia was demonstrated in older patients [30].

### Clinical implication

In hypertension, diuretic-based antihypertensive therapy has been shown to reduce stroke, heart failure, all-cause mortality, and death from cardiovascular disease in studies of patients aged ≥80 years [50, 51]. The subanalysis of the SPRINT study in patients aged ≥75 years also showed that intensive BP treatment reduced the risk of cardiovascular events and death compared with standard treatment [52]. In older patients with hypertension, antihypertensive drug therapy may be aggressively used if lifestyle modification is insufficient to lower BP, regardless of age. The addition of esaxerenone in patients aged ≥65 years with inadequate response to basal antihypertensive agents alone is expected to have a stronger antihypertensive effect than diuretics, without raising any safety concerns. A previous study reported that a −2.5 mmHg reduction in morning home SBP contributes to a 3.5%–9.5% reduction in cardiovascular disease risk [53]. Therefore, the −3.0 mmHg reduction in SBP with esaxerenone versus trichlormethiazide observed in the present study indicates a clinically meaningful reduction. Furthermore, a previous study reported that the ARB/CCB combination was superior to an ARB/diuretic combination in patients with uncontrolled nocturnal hypertension [54]. Further research is needed to determine whether esaxerenone or ARBs are more effective as a second-line antihypertensive agent when used in combination with a CCB.

### Limitations

The study limitations are the same as those of the primary EXCITE-HT study [29, 30]. Basal antihypertensive agents were prescribed by physicians under real-world clinical conditions as appropriate for patients, and there may have been background bias. No adjustments were made for patient background characteristics or baseline BP values, no age group tests were conducted, and outcomes were not compared between patients with high vs low BMI. Although hypertensive patients with low BMI are encountered in clinical practice, the study population was limited to patients who were able to attend outpatient clinics. Therefore, bedridden patients and hypertensive patients with sarcopenia and low BMI were not included, potentially limiting the generalizability of the findings. Renin activity was not measured. Finally, in this study, the subgroup analysis was performed only for two age groups (<65 years and ≥65 years), though the JSH 2019 guidelines set different blood pressure targets for those <75 years and ≥75 years [22]. However, the currently underway ESCORT-HT study [55], which will evaluate the antihypertensive efficacy and safety of esaxerenone in older hypertensive patients with inadequate response to a single calcium channel blocker, is expected to shed light on this question.

## Conclusion

The non-inferiority of esaxerenone to trichlormethiazide in lowering morning home BP regardless of age was confirmed. In patients aged <65 years, esaxerenone achieved the pre-defined non-inferiority margin to trichlormethiazide in lowering both SBP and DBP, and in patients aged ≥65 years, esaxerenone was superior to trichlormethiazide in lowering SBP and non-inferior to trichlormethiazide in lowering DBP. Additionally, significant reductions from baseline were observed in bedtime home and office SBP/DBP in both age subgroups. There was no specific age-related effect of esaxerenone on serum potassium levels.

## Supplementary information

Supplementary information

## Data Availability

The anonymized data underlying the results presented in this manuscript may be made available to researchers upon submission of a reasonable request to the corresponding author. The decision to disclose the data will be made by the corresponding author and the funder, Daiichi Sankyo Co., Ltd. Data disclosure can be requested for 36 months from article publication.
