# Supplementary material for: Home blood pressure-lowering effect of esaxerenone vs trichlormethiazide for uncontrolled hypertension: a prespecified subanalysis of the EXCITE-HT randomized controlled study by age subgroup
Source: Hypertens Res. 2025 Mar 28;48(4):1586–98. doi: 10.1038/s41440-024-02078-8 (PMC11972961; doi:10.1038/s41440-024-02078-8)
Supplement: Supplementary file 1 — Supplementary information [file 41440_2024_2078_MOESM1_ESM.docx]

**Home blood pressure-lowering effect of esaxerenone vs trichlormethiazide for uncontrolled hypertension: a prespecified subanalysis of the EXCITE-HT randomized controlled study by age subgroup**

Kazuomi Kario, Hiroyuki Ohbayashi, Masami Hashimoto, Naoki Itabashi, Mitsutoshi Kato, Kazuaki Uchiyama, Kunio Hirano, Noriko Nakamura, Takahide Miyamoto, Hirotaka Nagashima, Hidenori Ishida, Yusuke Ebe, Tsuguru Hatta, Toshiki Fukui, Tomohiro Katsuya, Tatsuo Shimosawa, Takashi Taguchi, Ayumi Tanabe, and Mitsuru Ohishi, on behalf of the EXCITE-HT investigators

**Supplementary Table 1.** Baseline patient characteristics (per-protocol set)

| **Characteristics** | **<65 years** | | **≥65 years** | |
| --- | --- | --- | --- | --- |
|  | **Esaxerenone**  ***n* = 128** | **Trichlormethiazide**  ***n* = 133** | **Esaxerenone**  ***n* = 147** | **Trichlormethiazide**  ***n* = 157** |
| Sex, male | 71 (55.5) | 82 (61.7) | 72 (49.0) | 78 (49.7) |
| Age, years | 54.8 ± 6.8 | 54.0 ± 7.9 | 74.1 ± 6.1 | 73.9 ± 6.1 |
| Weight, kg | 73.04 ± 14.66 | 73.16 ± 14.08 | 59.01 ± 10.92 | 61.10 ± 11.10 |
| Body mass index, kg/m^2^ | 27.02 ± 4.64 | 26.86 ± 4.66 | 23.74 ± 2.94 | 24.41 ± 3.38 |
| Morning home SBP, mmHg | 138.4 ± 15.1 | 136.9 ± 12.5 | 142.7 ± 15.0 | 141.6 ± 13.3 |
| Morning home DBP, mmHg | 90.5 ± 9.8 | 90.1 ± 9.2 | 84.1 ± 8.7 | 83.6 ± 8.4 |
| Bedtime home SBP, mmHg | *n* = 124  134.4 ± 16.2 | 134.0 ± 13.6 | *n* = 137 136.3 ± 15.8 | *n* = 149 134.8 ± 14.4 |
| Bedtime home DBP, mmHg | *n* = 124  84.9 ± 10.7 | 85.4 ± 10.1 | *n* = 137  78.9 ± 9.6 | *n* = 149 77.7 ± 10.2 |
| Office SBP, mmHg | 141.0 ± 14.1 | 140.1 ± 15.3 | 145.8 ± 18.2 | 145.0 ± 14.7 |
| Office DBP, mmHg | 88.2 ± 10.8 | 88.3 ± 11.6 | 79.4 ± 10.9 | 79.3 ± 11.2 |
| NT-proBNP, pg/mL | *n* = 99  51.91 ± 102.01 | *n* = 116 53.80 ± 166.14 | *n* = 124 157.75 ± 410.94 | *n* = 137 109.29 ± 124.43 |
| <55 | 74 (74.7) | 93 (80.2) | 46 (37.1) | 47 (34.3) |
| 55 to <125 | 18 (18.2) | 17 (14.7) | 40 (32.3) | 55 (40.1) |
| ≥125 | 7 (7.1) | 6 (5.2) | 38 (30.6) | 35 (25.5) |
| UACR, mg/gCr | 106.98 ± 603.70 | 96.87 ± 563.19 | 126.40 ± 373.05 | 105.15 ± 255.49 |
| <30 | 91 (71.1) | 98 (73.7) | 87 (59.2) | 89 (56.7) |
| 30 to <300 | 32 (25.0) | 28 (21.1) | 47 (32.0) | 55 (35.0) |
| ≥300 | 5 (3.9) | 7 (5.3) | 13 (8.8) | 13 (8.3) |
| Serum potassium, mEq/L | *n* = 124 4.16 ± 0.36 | *n* = 127 4.18 ± 0.32 | *n* = 142 4.25 ± 0.33 | *n* = 151 4.24 ± 0.32 |
| Uric acid, mg/dL | 5.54 ± 1.39 | 5.66 ± 1.15 | 5.29 ± 1.16 | *n* = 156 5.19 ± 1.22 |
| eGFR_creat_, mL/min/1.73 m^2^ | 76.78 ± 16.04 | *n* = 131  77.27 ± 15.61 | 67.41 ± 14.47 | *n* = 156  67.82 ± 17.14 |
| Duration of hypertension, years | *n* = 79  3.85 ± 4.28 | *n* = 86 4.41 ± 4.13 | *n* = 96 6.48 ± 5.36 | *n* = 98 6.38 ± 5.57 |
| Complication | 123 (96.1) | 121 (91.0) | 143 (97.3) | 155 (98.7) |
| T2DM | 45 (35.2) | 49 (36.8) | 61 (41.5) | 66 (42.0) |
| Dyslipidemia | 81 (63.3) | 71 (53.4) | 95 (64.6) | 100 (63.7) |
| Hyperuricemia | 28 (21.9) | 32 (24.1) | 15 (10.2) | 12 (7.6) |
| Heart failure | 1 (0.8) | 3 (2.3) | 18 (12.2) | 13 (8.3) |
| Esaxerenone dose at baseline (initial dose), mg |  |  |  |  |
| 1.25 | 33 (25.8) | - | 71 (48.3) | - |
| 2.5 | 95 (74.2) | - | 76 (51.7) | - |
| Esaxerenone dose at EOT (last dose), mg |  |  |  |  |
| 1.25 | 14 (10.9) | - | 41 (27.9) | - |
| 2.5 | 78 (60.9) | - | 87 (59.2) | - |
| 5 | 36 (28.1) | - | 19 (12.9) | - |
| Trichlormethiazide dose at baseline (initial dose), mg |  |  |  |  |
| 0.25 | - | 2 (1.5) | - | 2 (1.3) |
| 0.5 | - | 10 (7.5) | - | 7 (4.5) |
| 1 | - | 117 (88.0) | - | 145 (92.4) |
| 2 | - | 4 (3.0) | - | 3 (1.9) |
| Trichlormethiazide dose at EOT (last dose), mg |  |  |  |  |
| 0.25 | - | 2 (1.5) | - | 0 |
| 0.5 | - | 7 (5.3) | - | 11 (7.0) |
| 1 | - | 109 (82.0) | - | 135 (86.0) |
| >1 to ≤2 | - | 13 (9.8) | - | 11 (7.0) |
| ≥3 | - | 2 (1.5) | - | 0 |
| Basal antihypertensive agent |  |  |  |  |
| Angiotensin receptor blocker | 48 (37.5) | 56 (42.1) | 60 (40.8) | 60 (38.2) |
| Calcium channel blocker | 80 (62.5) | 77 (57.9) | 87 (59.2) | 97 (61.8) |

Data are n (%) or mean ± standard deviation.

*DBP* diastolic blood pressure, *eGFR_creat_* creatinine-based estimated glomerular filtration rate, *EOT* end of treatment, *NT-proBNP* N-terminal pro-brain natriuretic peptide, *SBP* systolic blood pressure, *T2DM* type 2 diabetes mellitus, *UACR* urinary albumin-to-creatinine ratio.

**Supplementary** **Table 2.** Change from baseline in BP (full analysis set)

| **BP** | **<65 years** | | | | | | **≥65 years** | | | | | |
| --- | --- | --- | --- | --- | --- | --- | --- | --- | --- | --- | --- | --- |
|  | **Esaxerenone** | | | **Trichlormethiazide** | | | **Esaxerenone** | | | **Trichlormethiazide** | | |
|  | ***n*** | **SBP, mmHg** | **DBP, mmHg** | ***n*** | **SBP, mmHg** | **DBP, mmHg** | ***n*** | **SBP, mmHg** | **DBP, mmHg** | ***n*** | **SBP, mmHg** | **DBP, mmHg** |
| Morning home BP | 137 |  |  | 133 |  |  | 158 |  |  | 157 |  |  |
| Baseline | 137 | 137.9 ± 14.8 | 90.2 ± 9.7 | 133 | 136.9 ± 12.5 | 90.1 ± 9.2 | 158 | 142.0 ± 15.0 | 84.0 ± 8.7 | 157 | 141.6 ± 13.3 | 83.6 ± 8.4 |
| Week 12 | 130 | 128.6 ± 13.1 | 84.7 ± 9.1 | 131 | 129.3 ± 12.6 | 85.5 ± 9.2 | 153 | 127.5 ± 13.0 | 76.9 ± 8.2 | 154 | 130.7 ± 11.7 | 77.2 ± 8.1 |
| Change from baseline | 130 | −9.5 ± 10.6 | −5.7 ± 6.3 | 131 | −7.6 ± 8.0 | −4.6 ± 5.0 | 153 | −14.1 ± 9.9 | −7.0 ± 5.0 | 154 | −10.9 ± 9.7 | −6.3 ± 5.4 |
| LS mean change from baseline [95% CI] |  | −9.6 [−11.0, −8.1] | −5.7 [−6.7, −4.8] |  | −8.0 [−9.4, −6.5] | −4.7 [−5.7, −3.8] |  | −14.5 [−15.8, −13.1] | −7.3 [−8.0, −6.5] |  | −11.4 [−12.7, −10.0] | −6.7 [−7.4, −6.0] |
| Difference in LS mean change from baseline [95% CI] |  | −1.6 [−3.6, 0.5] | −1.0 [−2.3, 0.3] |  | - | - |  | −3.1 [−5.0, −1.2] | −0.6 [−1.6, 0.5] |  | - | - |
| EOT | 136 | 128.4 ± 13.1 | 84.5 ± 9.0 | 133 | 129.0 ± 12.7 | 85.4 ± 9.2 | 158 | 127.8 ± 13.3 | 77.0 ± 8.2 | 157 | 130.7 ± 11.7 | 77.3 ± 8.0 |
| Change from baseline | 136 | −9.4 ± 10.5 | −5.6 ± 6.3 | 133 | −7.8 ± 8.3 | −4.8 ± 5.2 | 158 | −14.2 ± 10.1 | −7.0 ± 5.2 | 157 | −11.0 ± 9.7 | −6.3 ± 5.4 |
| LS mean change from baseline [95% CI] |  | −9.5 [−10.9, −8.0] | −5.7 [−6.6, −4.8] |  | −8.2 [−9.7, −6.8] | −4.9 [−5.8, −4.0] |  | −14.6 [−15.9, −13.2] | −7.2 [−7.9, −6.5] |  | −11.5  [−12.9, −10.2] | −6.7  [−7.4, −6.0] |
| Difference in LS mean change from baseline [95% CI] |  | −1.3 [−3.3, 0.8] | −0.8  [−2.1, 0.5] |  | - | - |  | −3.0 [−4.9, −1.2] | −0.5 [−1.5, 0.5] |  | - | - |
| Bedtime home BP | 137 |  |  | 133 |  |  | 158 |  |  | 157 |  |  |
| Baseline | 133 | 133.7 ± 16.0 | 84.6 ± 10.6 | 133 | 134.0 ± 13.6 | 85.4 ± 10.1 | 148 | 135.5 ± 15.7 | 78.7 ± 9.5 | 149 | 134.8 ± 14.4 | 77.7 ± 10.2 |
| Week 12 | 130 | 124.9 ± 13.6 | 79.3 ± 9.7 | 131 | 126.2 ± 14.3 | 80.3 ± 9.3 | 150 | 122.8 ± 13.0 | 72.0 ± 8.9 | 152 | 125.4 ± 12.7 | 72.2 ± 9.1 |
| Change from baseline | 126 | −9.8 ± 10.1*** | −5.9 ± 6.2*** | 131 | −7.7 ± 9.0*** | −5.0 ± 6.4*** | 143 | −12.7 ± 10.7*** | −6.8 ± 6.0*** | 146 | −9.7 ± 10.9*** | −5.5 ± 6.4*** |
| EOT | 136 | 124.8 ± 13.6 | 79.2 ± 9.6 | 133 | 126.1 ± 14.3 | 80.3 ± 9.2 | 155 | 122.9 ± 13.3 | 72.1 ± 8.9 | 155 | 125.3 ± 12.7 | 72.2 ± 9.0 |
| Change from baseline | 132 | −9.4 ± 10.1*** | −5.6 ± 6.3*** | 133 | −7.9 ± 9.2*** | −5.1 ± 6.4*** | 148 | −12.6 ± 10.7*** | −6.7 ± 6.0*** | 149 | −9.8 ± 10.9*** | −5.5 ± 6.4*** |
| Office BP | 137 |  |  | 133 |  |  | 158 |  |  | 157 |  |  |
| Baseline | 137 | 141.2 ± 14.0 | 87.9 ± 10.7 | 133 | 140.1 ± 15.3 | 88.3 ± 11.6 | 158 | 146.1 ± 18.1 | 79.6 ± 11.0 | 157 | 145.0 ± 14.7 | 79.3 ± 11.2 |
| Week 12 | 131 | 129.6 ± 13.6 | 82.1 ± 9.5 | 131 | 131.4 ± 14.4 | 83.2 ± 11.5 | 152 | 131.6 ± 17.8 | 72.6 ± 11.3 | 153 | 134.5 ± 14.7 | 74.5 ± 11.1 |
| Change from baseline |  | −11.6 ± 13.2*** | −5.7 ± 8.8*** |  | −8.5 ± 13.0*** | −5.0 ± 9.5*** |  | −14.4 ± 13.0*** | −7.0 ± 8.8*** |  | −10.5 ± 12.6*** | −4.9 ± 8.1*** |
| EOT | 136 | 129.6 ± 13.6 | 82.1 ± 9.4 | 133 | 131.5 ± 14.8 | 83.1 ± 11.7 | 158 | 131.6 ± 17.4 | 72.7 ± 11.2 | 157 | 134.5 ± 14.7 | 74.6 ± 11.1 |
| Change from baseline |  | −11.4 ± 13.4*** | −5.7 ± 8.8*** |  | −8.6 ± 13.1*** | −5.2 ± 9.3*** |  | −14.4 ± 12.8*** | −7.0 ± 8.7*** |  | −10.5 ± 12.5*** | −4.7 ± 8.0*** |

Data are mean ± SD.

***P <0.0001 versus baseline, paired *t*-test.

LS mean change were calculated for morning home BP at Week 12 and EOT.

LS mean change and 95% CIs were calculated using the analysis of covariance model, with morning home SBP/DBP change from baseline as the objective variable; treatment group as the explanatory variable; and baseline BP, baseline antihypertensive medication, and baseline age as covariates.

*BP* blood pressure, *CI* confidence interval, *DBP* diastolic blood pressure, *EOT* end of treatment, *LS* least squares, *SBP* systolic blood pressure, *SD* standard deviation.

**Supplementary** **Table 3.** Change from baseline in BP (per-protocol set)

| **BP** | **<65 years** | | | | | | **≥65 years** | | | | | |
| --- | --- | --- | --- | --- | --- | --- | --- | --- | --- | --- | --- | --- |
|  | **Esaxerenone** | | | **Trichlormethiazide** | | | **Esaxerenone** | | | **Trichlormethiazide** | | |
|  | ***n*** | **SBP, mmHg** | **DBP, mmHg** | ***n*** | **SBP, mmHg** | **DBP, mmHg** | ***n*** | **SBP, mmHg** | **DBP, mmHg** | ***n*** | **SBP, mmHg** | **DBP, mmHg** |
| Morning home BP | 128 |  |  | 133 |  |  | 147 |  |  | 157 |  |  |
| Baseline | 128 | 138.4 ± 15.1 | 90.5 ± 9.8 | 133 | 136.9 ± 12.5 | 90.1 ± 9.2 | 147 | 142.7 ± 15.0 | 84.1 ± 8.7 | 157 | 141.6 ± 13.3 | 83.6 ± 8.4 |
| Week 12 | 121 | 128.7 ± 13.4 | 84.7 ± 9.3 | 131 | 129.3 ± 12.6 | 85.5 ± 9.2 | 143 | 127.8 ± 12.9 | 77.1 ± 8.3 | 154 | 130.7 ± 11.7 | 77.2 ± 8.1 |
| Change from baseline | 121 | −10.0 ± 10.5 | −6.0 ± 6.2 | 131 | −7.6 ± 8.0 | −4.6 ± 5.0 | 143 | −14.3 ± 9.8 | −7.0 ± 5.1 | 154 | −10.9 ± 9.7 | −6.3 ± 5.4 |
| LS mean change from baseline [95% CI] |  | −10.1  [−11.6, −8.6] | −6.1  [−7.1, −5.2] |  | −8.1  [−9.5, −6.6] | −4.8  [−5.7, −3.9] |  | −14.5  [−15.9, −13.2] | −7.2  [−7.9, −6.4] |  | −11.5  [−12.8, −10.1] | −6.7  [−7.4, −6.0] |
| Difference in LS mean change from baseline [95% CI] |  | −2.0  [−4.1, 0.0] | −1.3  [−2.6, 0.0] |  | - | - |  | −3.1  [−5.0, −1.2] | −0.5  [−1.5, 0.6] |  | - | - |
| EOT | 127 | 128.5 ± 13.4 | 84.6 ± 9.2 | 133 | 129.0 ± 12.7 | 85.4 ± 9.2 | 147 | 128.3 ± 13.1 | 77.2 ± 8.3 | 157 | 130.7 ± 11.7 | 77.3 ± 8.0 |
| Change from baseline | 127 | −9.8 ± 10.5 | −5.9 ± 6.2 | 133 | −7.8 ± 8.3 | −4.8 ± 5.2 | 147 | −14.4 ± 10.1 | −6.9 ± 5.3 | 157 | −11.0 ± 9.7 | −6.3 ± 5.4 |
| LS mean change from baseline [95% CI] |  | −10.0  [−11.5, −8.5] | −6.0  [−7.0, −5.1] |  | −8.3  [−9.7, −6.8] | −4.9  [−5.9, −4.0] |  | −14.6  [−16.0, −13.3] | −7.1  [−7.8, −6.3] |  | −11.6  [−13.0, −10.3] | −6.7  [−7.5, −6.0] |
| Difference in LS mean change from baseline [95% CI] |  | −1.7  [−3.8, 0.4] | −1.1  [−2.4, 0.2] |  | - | - |  | −3.0  [−4.9, −1.1] | −0.4  [−1.4, 0.7] |  | - | - |
| Bedtime home BP | 128 |  |  | 133 |  |  | 147 |  |  | 157 |  |  |
| Baseline | 124 | 134.4 ± 16.2 | 84.9 ± 10.7 | 133 | 134.0 ± 13.6 | 85.4 ± 10.1 | 137 | 136.3 ± 15.8 | 78.9 ± 9.6 | 149 | 134.8 ± 14.4 | 77.7 ± 10.2 |
| Week 12 | 121 | 125.4 ± 13.8 | 79.6 ± 9.8 | 131 | 126.2 ± 14.3 | 80.3 ± 9.3 | 140 | 122.9 ± 13.1 | 72.2 ± 8.9 | 152 | 125.4 ± 12.7 | 72.2 ± 9.1 |
| Change from baseline | 117 | −10.0 ± 9.9*** | −6.0 ± 5.8*** | 131 | −7.7 ± 9.0*** | −5.0 ± 6.4*** | 133 | −13.2 ± 10.7*** | −6.8 ± 6.0*** | 146 | −9.7 ± 10.9*** | −5.5 ± 6.4*** |
| EOT | 127 | 125.3 ± 13.7 | 79.4 ± 9.6 | 133 | 126.1 ± 14.3 | 80.3 ± 9.2 | 144 | 123.2 ± 13.4 | 72.2 ± 8.9 | 155 | 125.3 ± 12.7 | 72.2 ± 9.0 |
| Change from baseline | 123 | −9.6 ± 10.0*** | −5.8 ± 5.9*** | 133 | −7.9 ± 9.2*** | −5.1 ± 6.4*** | 137 | −13.1 ± 10.6*** | −6.7 ± 6.0*** | 149 | −9.8 ± 10.9*** | −5.5 ± 6.4*** |
| Office BP | 128 |  |  | 133 |  |  | 147 |  |  | 157 |  |  |
| Baseline | 128 | 141.0 ± 14.1 | 88.2 ± 10.8 | 133 | 140.1 ± 15.3 | 88.3 ± 11.6 | 147 | 145.8 ± 18.2 | 79.4 ± 10.9 | 157 | 145.0 ± 14.7 | 79.3 ± 11.2 |
| Week 12 | 122 | 129.8 ± 13.6 | 82.4 ± 9.5 | 131 | 131.4 ± 14.4 | 83.2 ± 11.5 | 142 | 131.6 ± 18.3 | 72.5 ± 11.4 | 153 | 134.5 ± 14.7 | 74.5 ± 11.1 |
| Change from baseline | 122 | −11.1 ± 13.3*** | −5.7 ± 8.9*** | 131 | −8.5 ± 13.0*** | −5.0 ± 9.5*** | 142 | −14.2 ± 12.7*** | −6.9 ± 8.9*** | 153 | −10.5 ± 12.6*** | −4.9 ± 8.1*** |
| EOT | 127 | 129.8 ± 13.7 | 82.4 ± 9.4 | 133 | 131.5 ± 14.8 | 83.1 ± 11.7 | 147 | 131.5 ± 18.0 | 72.4 ± 11.3 | 157 | 134.5 ± 14.7 | 74.6 ± 11.1 |
| Change from baseline | 127 | −11.0 ± 13.5*** | −5.6 ± 9.0*** | 133 | −8.6 ± 13.1*** | −5.2 ± 9.3*** | 147 | −14.3 ± 12.5*** | −6.9 ± 8.8*** | 157 | −10.5 ± 12.5*** | −4.7 ± 8.0*** |

Data are mean ± SD.

***P <0.0001 versus baseline, paired *t*-test.

LS mean change were calculated for morning home BP at Week 12 and EOT.

LS mean change and 95% CIs were calculated using the analysis of covariance model, with morning home SBP/DBP change from baseline as the objective variable; treatment group as the explanatory variable; and baseline BP, baseline antihypertensive medication, and baseline age as covariates.

*BP* blood pressure, *CI* confidence interval, *DBP* diastolic blood pressure, *EOT* end of treatment, *LS* least squares, *SBP* systolic blood pressure, *SD* standard deviation.

**Supplementary** **Table 4.** Change in UACR and NT-proBNP from baseline to Week 12 (full analysis set)

| **Variables** | **<65 years** | | | | **≥65 years** | | | |
| --- | --- | --- | --- | --- | --- | --- | --- | --- |
|  | **Esaxerenone** | | **Trichlormethiazide** | | **Esaxerenone** | | **Trichlormethiazide** | |
| **UACR, mg/gCr** | ***n*** |  | ***n*** |  | ***n*** |  | ***n*** |  |
| Baseline | 137 | 103.50 ± 583.89 | 133 | 96.87 ± 563.19 | 158 | 127.33 ± 374.21 | 157 | 105.15 ± 255.49 |
| Week 4 | 137 | 84.82 ± 564.85 | 133 | 69.06 ± 383.48 | 158 | 71.82 ± 198.36 | 157 | 71.11 ± 271.09 |
| Change from baseline | 137 | −18.68 ± 88.82* | 133 | −27.82 ± 204.47 | 158 | −55.51 ± 231.12* | 157 | −34.04 ± 179.03* |
| Percentage change in geometric mean from baseline [95% CI] |  | −30.0  [−38.1, −20.9]*** |  | −23.7  [−32.8, −13.4]*** |  | −35.5  [−42.6, −27.5]*** |  | −33.1  [−40.7, −24.5]*** |
| Week 8 | 133 | 32.65 ± 109.24 | 131 | 62.21 ± 335.96 | 154 | 65.09 ± 190.72 | 155 | 49.85 ± 142.62 |
| Change from baseline | 133 | −17.93 ± 92.47* | 131 | −35.43 ± 258.92 | 154 | −49.68 ± 181.13** | 155 | −56.55 ± 171.42*** |
| Percentage change in geometric mean from baseline [95% CI] |  | −34.0  [−42.6, −24.2]*** |  | −27.6  [−36.4, −17.6]*** |  | −41.8  [−48.8, −33.9]*** |  | −41.8  [−48.3, −34.6]*** |
| Week 12 | 131 | 43.61 ± 181.44 | 131 | 53.99 ± 315.48 | 152 | 60.70 ± 266.15 | 153 | 54.68 ± 201.16 |
| Change from baseline | 131 | −6.94 ± 183.42 | 131 | −43.65 ± 266.54 | 152 | −48.33 ± 169.08** | 153 | −52.55 ± 175.70** |
| Percentage change in geometric mean from baseline [95% CI] |  | −28.3  [−38.8, −15.9]*** |  | −38.1  [−45.6, −29.6]*** |  | −46.8  [−53.9, −38.7]*** |  | −45.0  [−51.6, −37.4]*** |
| **NT-proBNP, pg/mL** |  |  |  |  |  |  |  |  |
| Baseline | 104 | 51.75 ± 99.88 | 116 | 53.80 ± 166.14 | 133 | 154.64 ± 397.27 | 137 | 109.29 ± 124.43 |
| Week 12 | 96 | 36.79 ± 45.25 | 108 | 50.81 ± 114.42 | 124 | 109.03 ± 148.34 | 133 | 95.45 ± 121.15 |
| Change from baseline | 96 | −14.25 ± 78.21 | 106 | −4.29 ± 102.45 | 124 | −47.68 ± 317.18 | 132 | −13.73 ± 81.49 |

Data are geometric mean ± standard deviation.

*P <0.05, **P <0.01, ***P <0.001 versus baseline, paired *t*-test.

For UACR, P*-*values are only presented for percentage change in geometric mean from baseline.

*CI* confidence interval, *UACR* urinary albumin-to-creatinine ratio, *NT-proBNP* N-terminal pro-brain natriuretic peptide.

**Supplementary** **Table 5.** Change in UACR and NT-proBNP from baseline to Week 12 (per-protocol set)

| **Variables** | **<65 years** | | | | **≥65 years** | | | |
| --- | --- | --- | --- | --- | --- | --- | --- | --- |
|  | **Esaxerenone** | | **Trichlormethiazide** | | **Esaxerenone** | | **Trichlormethiazide** | |
| **UACR, mg/gCr** | ***n*** |  | ***n*** |  | ***n*** |  | ***n*** |  |
| Baseline | 128 | 106.98 ± 603.70 | 133 | 96.87 ± 563.19 | 147 | 126.40 ± 373.05 | 157 | 105.15 ± 255.49 |
| Week 4 | 128 | 88.26 ± 584.19 | 133 | 69.06 ± 383.48 | 147 | 72.86 ± 201.60 | 157 | 71.11 ± 271.09 |
| Change from baseline | 128 | −18.73 ± 90.71* | 133 | −27.82 ± 204.47 | 147 | −53.54 ± 230.23* | 157 | −34.04 ± 179.03* |
| Percentage change in geometric mean from baseline [95% CI] |  | −28.9  [−37.5, −19.2]*** |  | −23.7  [−32.8, −13.4]*** |  | −35.1  [−42.7, −26.5]*** |  | −33.1  [−40.7, −24.5]*** |
| Week 8 | 124 | 33.37 ± 112.83 | 131 | 62.21 ± 335.96 | 144 | 66.60 ± 195.81 | 155 | 49.85 ± 142.62 |
| Change from baseline | 124 | −16.96 ± 94.02* | 131 | −35.43 ± 258.92 | 144 | −45.59 ± 166.58* | 155 | −56.55 ± 171.42*** |
| Percentage change in geometric mean from baseline [95% CI] |  | −32.3  [−41.4, −21.7]*** |  | −27.6  [−36.4, −17.6]*** |  | −42.6  [−49.8, −34.4]*** |  | −41.8  [−48.3, −34.6]*** |
| Week 12 | 122 | 44.69 ± 187.77 | 131 | 53.99 ± 315.48 | 142 | 60.48 ± 271.97 | 153 | 54.68 ± 201.16 |
| Change from baseline | 122 | −5.61 ± 189.52 | 131 | −43.65 ± 266.54 | 142 | −45.52 ± 162.23* | 153 | −52.55 ± 175.70** |
| Percentage change in geometric mean from baseline [95% CI] |  | −27.0  [−38.3, −13.7]** |  | −38.1  [−45.6, −29.6]*** |  | −46.8  [−54.3, −38.2]*** |  | −45.0  [−51.6, −37.4]*** |
| **NT-proBNP, pg/mL** |  |  |  |  |  |  |  |  |
| Baseline | 99 | 51.91 ± 102.01 | 116 | 53.80 ± 166.14 | 124 | 157.75 ± 410.94 | 137 | 109.29 ± 124.43 |
| Week 12 | 91 | 37.30 ± 46.15 | 108 | 50.81 ± 114.42 | 116 | 109.41 ± 151.76 | 133 | 95.45 ± 121.15 |
| Change from baseline | 91 | −13.88 ± 80.21 | 106 | −4.29 ± 102.45 | 116 | −50.80 ± 327.66 | 132 | −13.73 ± 81.49 |

Data are geometric mean ± standard deviation.

*P <0.05, **P <0.01, ***P <0.001 versus baseline, paired *t*-test.

For UACR, P*-*values are only presented for percentage change in geometric mean from baseline.

*CI* confidence interval, *UACR* urinary albumin-to-creatinine ratio, *NT-proBNP* N-terminal pro-brain natriuretic peptide.

**Supplementary** **Table 6.** Change in serum potassium and eGFR_creat_ from baseline to Week 12 (safety analysis set)

|  | **<65 years** | | | | **≥65 years** | | | |
| --- | --- | --- | --- | --- | --- | --- | --- | --- |
|  | **Esaxerenone *n* = 138** | | **Trichlormethiazide *n* = 134** | | **Esaxerenone *n* = 164** | | **Trichlormethiazide  *n* = 164** | |
|  | ***n*** | **Mean ± SD** | ***n*** | **Mean ± SD** | ***n*** | **Mean ± SD** | ***n*** | **Mean ± SD** |
| **Serum potassium (mEq/L)** |  |  |  |  |  |  |  |  |
| Baseline | 134 | 4.16 ± 0.36 | 127 | 4.18 ± 0.32 | 159 | 4.25 ± 0.33 | 158 | 4.23 ± 0.35 |
| Week 2 | 132 | 4.32 ± 0.37 | 127 | 4.05 ± 0.37 | 153 | 4.39 ± 0.33 | 155 | 4.12 ± 0.38 |
| Change from baseline | 132 | 0.15 ± 0.35 | 126 | −0.13 ± 0.30 | 152 | 0.15 ± 0.36 | 155 | −0.12 ± 0.33 |
| Week 4 | 134 | 4.21 ± 0.38 | 127 | 4.06 ± 0.37 | 152 | 4.34 ± 0.36 | 152 | 4.12 ± 0.34 |
| Change from baseline | 133 | 0.04 ± 0.37 | 126 | −0.11 ± 0.34 | 151 | 0.09 ± 0.37 | 152 | −0.11 ± 0.35 |
| Week 8 | 129 | 4.21 ± 0.35 | 126 | 3.97 ± 0.33 | 150 | 4.37 ± 0.37 | 150 | 4.12 ± 0.35 |
| Change from baseline | 129 | 0.06 ± 0.36 | 125 | −0.20 ± 0.32 | 149 | 0.12 ± 0.39 | 150 | −0.12 ± 0.35 |
| Week 12 | 127 | 4.14 ± 0.34 | 125 | 3.94 ± 0.36 | 148 | 4.32 ± 0.32 | 147 | 4.04 ± 0.34 |
| Change from baseline | 127 | −0.01 ± 0.36 | 125 | −0.24 ± 0.33 | 147 | 0.08 ± 0.34 | 147 | −0.20 ± 0.36 |
| **eGFR_creat_ (mL/min/1.73m^2^)** |  |  |  |  |  |  |  |  |
| Baseline | 138 | 76.70 ± 15.83 | 132 | 77.17 ± 15.59 | 164 | 67.10 ± 14.31 | 163 | 67.73 ± 17.18 |
| Week 2 | 137 | 71.80 ± 15.34 | 134 | 73.60 ± 15.88 | 158 | 61.49 ± 12.45 | 161 | 64.79 ± 14.85 |
| Change from baseline | 137 | −4.80 ± 8.23 | 132 | −3.48 ± 8.50 | 158 | −5.56 ± 7.94 | 160 | −3.03 ± 9.13 |
| Week 4 | 137 | 72.78 ± 15.06 | 133 | 72.88 ± 15.47 | 158 | 60.89 ± 12.78 | 158 | 64.41 ± 14.98 |
| Change from baseline | 137 | −3.82 ± 7.46 | 131 | −4.00 ± 8.29 | 158 | −6.18 ± 8.71 | 157 | −3.62 ± 9.22 |
| Week 8 | 133 | 71.79 ± 16.13 | 132 | 72.07 ± 15.46 | 154 | 59.70 ± 12.57 | 156 | 64.04 ± 15.75 |
| Change from baseline | 133 | −4.91 ± 8.46 | 130 | −5.00 ± 9.71 | 154 | −7.26 ± 8.93 | 155 | −4.05 ± 9.60 |
| Week 12 | 131 | 70.53 ± 15.01 | 131 | 72.53 ± 15.17 | 152 | 59.19 ± 12.38 | 153 | 63.41 ± 14.29 |
| Change from baseline | 131 | −6.00 ± 8.08 | 129 | −4.61 ± 8.98 | 152 | −8.02 ± 8.96 | 152 | −4.73 ± 10.54 |

P-values were not calculated.

*eGFR_creat_* creatinine-based estimated glomerular filtration rate*, SD* standard deviation.

**Supplementary** **Table 7.** Incidence of serum potassium level <3.5, ≥5.5, and ≥6.0 mEq/L (safety analysis set)

| **Serum potassium level** | **<65 years** | | **≥65 years** | |
| --- | --- | --- | --- | --- |
|  | **Esaxerenone**  ***n* = 138** | **Trichlormethiazide**  ***n* = 134** | **Esaxerenone**  ***n* = 164** | **Trichlormethiazide**  ***n* = 164** |
| Serum potassium <3.5 mEq/L | 6/135 (4.4) [1.6, 9.4] | 18/129 (14.0) [8.5, 21.2] | 3/160 (1.9) [0.4, 5.4] | 15/158 (9.5) [5.4, 15.2] |
| Serum potassium ≥5.5 mEq/L | 3/135 (2.2) [0.5, 6.4] | 1/129 (0.8) [0.0, 4.2] | 3/160 (1.9) [0.4, 5.4] | 1/158 (0.6) [0.0, 3.5] |
| Serum potassium ≥6.0 mEq/L | 0/135 [0.0, 2.7] | 0/129 [0.0, 2.8] | 0/160 [0.0, 2.3] | 0/158 [0.0, 2.3] |

Data are n/N (%) [95% CI].

*CI* confidence interval.

**Supplementary** **Table 8.** Incidence of UA level >7.0 mg/dL (safety analysis set)

| **UA level** | **<65 years** | | **≥65 years** | |
| --- | --- | --- | --- | --- |
|  | **Esaxerenone**  ***n* = 138** | **Trichlormethiazide**  ***n* = 134** | **Esaxerenone**  ***n* = 164** | **Trichlormethiazide**  ***n* = 164** |
| UA >7.0 mg/dL | 46/138 (33.3) [25.5, 41.9] | 58/134 (43.3) [34.8, 52.1] | 37/164 (22.6) [16.4, 29.7] | 45/164 (27.4) [20.8, 34.9] |

Data are n/N (%) [95% CI].

*CI* confidence interval, *UA* uric acid.
